# Enhancing C2 pedicle screw placement in Hangman’s fractures: a comparative study of navigation and 3D printed guidance techniques

**DOI:** 10.3389/fsurg.2025.1661304

**Published:** 2025-11-13

**Authors:** Rao Ruiqiang, Li Yi, Xiao Haiyan, Wang Minggui, Liao Yixiu, Shang Song

**Affiliations:** 1 Department of Orthopedics, Chongqing University Fuling Hospital, Chongqing, China; 2 Department of Orthopedics, Sichuan Gem Flower Hospital, Chengdu, Sichuan, China; 3 Department of Ultrasound, Chongqing University Fuling Hospital, Chongqing, China

**Keywords:** C2 pedicle screw placement, Hangman's fractures, navigation, 3D printed guidance techniques, comparative study

## Abstract

**Objective:**

This retrospective study aimed to analyze the accuracy and safety of pedicle screw placement in upper cervical spine surgery for Hangman's fractures, particularly in cases with high-riding vertebral artery (HRVA) at C2, using navigation technology and 3D-printed patient-specific templates.

**Methods:**

We included 33 patients with Hangman's fractures who underwent posterior cervical fixation with bone graft fusion or non-bone graft fusion surgery between January 2013 and January 2023. Patients were divided into two groups based on the intraoperative pedicle screw placement method: Group A (*n* = 15) utilized a navigation system, and Group B (*n* = 18) used a 3D printed navigation template. Clinical and radiological data were collected and compared.

**Results:**

One hundred and forty-four screws (66 navigated, 78 3D-printed templates) were inserted without vertebral artery, spinal cord, or nerve injury. Operative time per screw: Group A 24.0 ± 5.6 min vs. Group B 18.7 ± 4.1 min; mean difference 5.3 min (22% reduction; 95% CI −9.1 to −1.5; *P* = 0.018). Intra-operative blood loss:152 ± 29 ml vs. 105 ± 19 mL; mean difference 47 mL (95% CI −66 to −19; *P* = 0.001). Overall accuracy (Grade 0 + 1) was 98.5% (65/66) vs. 98.7% (77/78) (odds ratio 1.05; 95% CI 0.11–10.2; *P* = 0.97). In the Type-III fracture subgroup (8 screws per group), templates achieved 100% accuracy vs. 87.5% with navigation (*P* = 0.032) and lower cortical penetration (12.5% vs. 37.5%; *P* = 0.008). Post-operative VAS and NDI improved comparably (all inter-group *P* > 0.05). At 24-month follow-up, all fractures had healed; C2/3 fusion rates did not differ (facet fusion 100% vs. 100%; inter-body fusion 39% vs. 39%; *P* > 0.99).

**Conclusion:**

The use of 3D-printed patient-specific templates in upper cervical spine surgery for Hangman's fractures, especially in the presence of HRVA, is effective and safe. It offers comparable accuracy to navigation-assisted surgery while reducing operative time and blood loss. This approach may be particularly beneficial in minimizing the risk of vertebral artery injury and optimizing surgical outcomes. Compared to navigation technology, it can effectively improve the accuracy of screw placement in extremely unstable fractures.

## 1 Introduction

Hangman's fracture is a traumatic spondylolisthesis of the axis, typically caused by sudden flexion-extension or axial compression forces, resulting in fractures between the superior and inferior articular processes (1). It is often associated with injuries to the anterior and posterior longitudinal ligaments, adjacent intervertebral discs, and axis dislocation, commonly caused by traffic accidents and falls from heights. This fracture is frequently accompanied by spinal cord compression, which can be fatal. Studies have classified such fractures into three types and five categories (2, 3). Clinically, Type II and Type IIa Hangman's fractures are considered unstable, and complications such as pseudoarthrosis, cervical instability, and chronic refractory neck pain may arise from conservative treatment, thus surgical intervention is often preferred (4). Biomechanical studies have shown that C2 pedicle screws have greater holding power and pull-out strength, making the internal fixation system constructed with C2 pedicle screws ideally strong (5–7). However, the C2 structure is complex and adjacent to important blood vessels and nerves. Particularly, the anatomical variation rate of the vertebral artery is high. Studies have found that the incidence of high-riding vertebral artery deformity is as high as 14.4%–33.9% (8–10). When a high-riding vertebral artery occurs, changes in the height and thickness of the C2 isthmus significantly increase the difficulty of placing a pedicle screw, requiring clinicians to have a high level of accuracy to avoid vascular injury. The risk of arterial injury during screw placement in cases of high-riding vertebral artery is extremely high, which can lead to a series of serious complications, such as intraoperative bleeding, massive blood loss, neurological impairment, and stroke (10). There is even a risk of brain stem infarction, vertebral-basilar artery insufficiency, and even death (11). Navigation technology can accurately place screws and reduce surgical risks, but the cervical spine is flexible, and navigation defocus often occurs during surgery (12). There are also issues with soft tissue obstruction leading to inward migration of the navigated screw placement points and fractures of the pedicle (13). With the development of digital orthopedics, 3D printing technology has gradually been applied to upper cervical spine screw placement to improve the accuracy of screw placement. The use of 3D-printed patient-specific templates in scoliosis correction surgery with pedicle screw fixation offers the advantages of optimizing preoperative planning, providing individualized and precise treatment, reducing screw placement difficulty, and improving accuracy. Preoperatively, precise specifications of planned screws, design of entry angles, and screw depths can be calculated with computer assistance. Simulated screw placement operations can enhance the surgeon's proficiency and provide a preview of key areas and potential challenges, reducing the risk of spinal cord or nerve injury during screw placement (14). 3D-printed patient-specific templates have excellent compatibility with the surgery segments, allowing for templates directly onto the vertebral lamina surface, which facilitates accurate guidance for screw placement, reducing the need for secondary screw placement attempts and lowering difficulty and risks (15). After screw placement, only one confirmatory fluoroscopy is needed, greatly shortening screw placement time and reducing x-ray exposure and blood loss (16). While navigation systems improve accuracy, their utility in extremely unstable Type III fractures remains limited. This study is the first to compare 3D-printed templates vs. navigation in HRVA patients. We hypothesised that 3D-printed patient-specific templates would achieve non-inferior accuracy compared with intra-operative navigation for C2 pedicle screw placement in Hangman's fractures with high-riding vertebral artery, while reducing operative time, fluoroscopy duration and complication rates. The primary comparative end-points were (1) screw accuracy graded by post-operative CT (Neo classification) and (2) operative time per screw; secondary end-points included intra-operative blood loss, fluoroscopy time, cortical penetration rate and procedure-related complications.

## 2 Materials and methods

### 2.1 General data

A retrospective analysis was conducted on patients with Hangman's fractures treated in our department from January 2013 to January 2023. Inclusion criteria: (1) Type II, IIa, and III fractures in the Levine-Edwards classification; (2) High-riding C2 vertebral artery: high-riding vertebral artery as a height of the isthmus internal (from the top of the vertebral artery foramen to the upper articular surface) less than 2 mm, or a thickness of the isthmus (measured at the midpoint of the atlantoaxial joint) less than 5 mm, or a combination of both (10); (3) Posterior cervical fixation with bone graft fusion or non-bone graft fusion surgery; (4) Intraoperative use of navigation or 3D printed navigation guide; (5) Follow-up for more than 2 years with complete clinical and imaging data. Exclusion criteria: (1) Severe intervertebral disc injury, spinal cord compression; (2) Additional anterior surgery or one-stage combined anterior and posterior surgery; (3) History of cervical spine surgery; (4) Local inflammation, infection, tuberculosis, tumor of the cervical spine; (5) Severe internal diseases, severe osteoporosis.

Assuming an expected 15% absolute difference in screw accuracy (90% vs. 75%) between 3-D template and navigation, *α* = 0.05 (two-sided), *β* = 0.20, we required 29 evaluable screws per arm. Inflating by 10% for loss to follow-up, we aimed to enrol at least 30 patients (≈60 screws) overall. A total of 33 patients met the above inclusion and exclusion criteria. Eligible patients were randomly allocated (1:1) using computer-generated permuted block randomisation (block size = 6) produced by an independent statistician. The 33 patients were numbered and randomly assigned to Groups A and B using a computer. Group A, 15 cases, received posterior fixation with navigation system-guided screw placement; Group B, 18 cases, received posterior fixation with 3D printed navigation template-guided screw placement. Due to the high risk of navigation drift caused by cervical spine elastic displacement during surgery for Type III fractures, we conducted a subgroup analysis of Type III cases in both Groups A and B. The grouping criterion was the presence of C2 vertebral body translation >3.5 mm and angulation >11° on lateral x-ray or CT, along with disappearance of the C2–C3 facet joint space or the “double facet sign.” The general data of patients in the two groups are shown in Table 1, and there were no statistical differences in gender, age, Levine-Edwards classification (*P* > 0.05).

**Table 1:** Comparison of general information between the two groups.

Characteristic	Group A (*n* = 15)	Group B (*n* = 18)	Statistical Value	*P* value
Age (years)	45.62 ± 15.15	45.57 ± 16.72	*t* = −0.131	0.425
Gender (*n*)			x^2^ = 1.52	0.554
Male	12	14		
Female	3	4		
Levine-Edwards classification			x^2^ = 1.63	0.322
Type II	6	7		
Type IIa	6	8		
Type III	3	3		
High-riding Vertebral Artery (*n*)	15	18		
Internal Height of the Isthmus (mm)	1.75 ± 0.23	1.77 ± 0.28	*t* = −0.321	0.918
Thickness of the Isthmus (mm)	4.41 ± 0.65	4.37 ± 0.73	*t* = −0.263	0.887

All experiments involving human subjects and the use of human tissue samples were conducted in accordance with the ethical standards of the institutional and national research committee and with the 1964 Helsinki declaration and its later amendments or comparable ethical standards. The experimental protocols were approved by the Ethics Committee of The Affiliated Fuling Hospital of Chongqing University (IRB number/approval date: 202210125/October 6, 2022). Informed consent was obtained from all individual participants included in the study and/or their legal guardian(s).

### 2.2 Preoperative preparation

After admission, all patients underwent cervical spine anteroposterior and lateral views, three-dimensional CT reconstruction, and cervical spine MRI to clarify the diameter and direction of the C2 pedicle, the degree of fracture displacement, and the condition of intervertebral discs and anterior longitudinal ligament injury. When pre-operative CT showed isthmus width <4 mm or internal height <1.5 mm (HRVA), a C2 laminar screw trajectory was pre-planned to avoid vertebral artery injury. Cervical CTA was performed to understand the variation of the vertebral artery. After admission, skull traction was performed under local anesthesia, with a traction weight of 3–5 Kg. Cervical spine x-ray was performed on the third day after admission to clarify the reduction situation.

### 2.3 Guide plate preparation

Preoperative spinal CT scans were performed on patients using a Siemens SOMATOM Drive CT scanner, with a slice thickness of 0.24 mm, and the scanned images were saved in Dicom format. The Dicom format files were imported into the 3D image generation and editing software Mimics21.0 (Materialise, Belgium) to generate three-dimensional models of the target vertebrae. In the computer-aided design software Creo 2.0 (PTC, USA), a cylindrical model with a diameter of 2.0 mm was used to simulate pedicle screws, and screws were placed parallel to the pedicles on both sides in the direction parallel to the pedicle. The position relationship between the screw trajectory and the pedicle was observed in the transverse, sagittal, and coronal views of the 3D interface, and the screw trajectory was finely adjusted to ensure that the trajectory did not penetrate the cortical bone of the pedicle and that the fine pedicles did not penetrate the inner cortical bone. If screws cannot be placed through the pedicle, a laminar screw fixation plan is designed for the guide plate. Taking a single vertebra as a unit, a hollow guide tube with a length of 30 mm, an inner diameter of 2.0 mm, and an outer diameter of 3.5 mm was made on the surface of the vertebral arch using the axis of the cylindrical model as the axis. After designing the navigation template screw pathways and reference points for each vertebral body, the interference portion between the navigation template and the three-dimensional model of the vertebral body was removed by Boolean difference to form the positioning surface of the navigation template. The template file was imported into a Forml + 3D printer (Formlabs, USA), and the pedicle screw navigation template was printed using photosensitive resin material(Formlabs Standard Resin tensile strength 60 MPa, flexural modulus 1.6 GPa). Preoperative surgical simulation was performed on the 3D-printed model of the cervical vertebra, A pilot drill was performed on five 3D print C2 models; all drill paths were contained within the pedicle cortex (100% Grade 0). And channels were created through the pedicles using the template channels. Surface inspection confirmed that the channels were located within the pedicles and did not breach the cortical bone of the pedicles. each template was fitted to the corresponding 3-D-reconstructed vertebra; only templates with <0.3 mm deviation on 3-D superimposition analysis were accepted. External validation confirmed the accuracy of the 3D-printed navigation template. After confirming the feasibility of the plan, the template was sterilized using ethylene oxide for later use.Mean template manufacturing time (DICOM import to ethylene-oxide sterilisation) was 21.3 ± 2.7 h; emergency night-shift workflow allowed < 18 h when necessary.

### 2.4 Surgical method

All patients underwent general anesthesia, prone position, and skull traction with a traction bow to fix the head; the surgical area was marked, routinely disinfected, and draped. The skin and subcutaneous tissue were sequentially incised, and the paravertebral muscles on both sides were stripped to expose the posterior arch of the atlas, the lamina and articular processes of the axis, and the spinous process and articular processes of C3. Group A: After the surgical exposure was completed, the navigation reference frame was fixed to the spinous process of C3, and a three-dimensional scan and reconstruction of the vertebrae were performed using the O-arm navigation system (Medtronic, Minneapolis, MN, USA) with StealthStation software (v. 3.0). After importing into the operating system for navigation registration, the navigation trephine was used to make an opening at the appropriate position under the guidance of navigation, and then the navigation cone was used to gradually expand the screws channel. The navigation probe was used to explore the screws channel, and after exploring the walls and front end of the screws channel without damage, the appropriate length of the pedicle screw (Kanghui) was placed. Before the formation of the second screws tunnel, the accuracy of navigation needs to be re-verified, because the cervical spine has large mobility, and slight operation can also cause displacement, so it is necessary to verify at any time. After the screw was placed, the position of the screw was confirmed by fluoroscopy until the position of the screw was adjusted to satisfaction. The appropriate length of the longitudinal connecting rod was installed, and the instrument was used to appropriately stretch and pull the reduction. Fluoroscopy showed that the dislocation of the axis was corrected, and the position of the internal fixation was good. The wound was thoroughly rinsed, hemostasis was performed, and a drainage tube was placed in the wound, and the wound was sutured layer by layer, and sterile dressing was applied for pressure dressing, and the neck brace was immobilized, and the operation was completed. Group B: The disinfected modified 3D printed guide plate was closely attached to the corresponding lateral mass, lamina, and posterior part of the spinous process of the atlas and axis, and it was observed whether the guide plate was closely attached to it. The assistant held the opposite side benchmark to fix the guide plate, and the operator drilled through the guide channel of the guide plate with a 2.0 mm diameter electric drill to break the cortical bone of the lamina for 10 mm. The hand drill was used to probe the pedicle screw channel through the guide channel, and the direction parallel to the guide benchmark was observed during the operation, and attention was paid to observe whether the benchmark direction and movement. After the drilling was completed, the probe was used to detect whether the inner wall of the screws channel was intact, and after confirmation, the guide plate was removed, the thread was tapped, and the pedicle screw was placed. After the screws was placed, the position of the screw was confirmed by fluoroscopy until the position of the screw was adjusted to satisfaction. The appropriate length of the longitudinal connecting rod was installed, and the instrument was used to appropriately stretch and pull the reduction. Fluoroscopy showed that the dislocation of the axis was corrected, and the position of the internal fixation was good. The wound was thoroughly rinsed, hemostasis was performed, and a drainage tube was placed in the wound, and the wound was sutured layer by layer, and sterile dressing was applied for pressure dressing, and the neck brace was immobilized, and the operation was completed. Patients in Groups A and B were strictly bedridden after surgery, with neck immobilization and axial turning; the drainage volume of the surgical area was observed. If it was less than 50 ml/24 h and there was no cerebrospinal fluid leakage, the incision drainage tube was removed; antibiotics were used for 24–48 h after surgery (for the elderly, malnourished, and diabetic patients, it was extended to 48 h), and if there were spinal cord nerve symptoms before surgery, steroids were used for 3–5 days. Three days after surgery, patients were advised to wear a neck brace and gradually get out of bed for activities.

### 2.5 Follow-up and evaluation indexes

Postoperative follow-up was conducted at 7 days and every 3 months after surgery, and cervical spine anteroposterior and lateral x-ray films and CT were reviewed to observe the internal fixation. The screws placement time and operation time of each group were recorded. Cervical spine CT was reviewed at 7 days after surgery, and the postoperative pedicle screw position was graded according to the Neo grading standard, and the screw placement satisfaction rate and cortical penetration rate were evaluated. The Japanese Orthopaedic Association (JOA) score and cervical dysfunction index (NDI) were used to evaluate the cervical nerve function of patients before and after surgery and at the last follow-up. The occurrence of complications was observed. The calculation method for screws placement time: For Group A, the time was recorded from the completion of exposure, the installation of the dynamic reference clamp, until the last pedicle screw was placed and the screw was confirmed to be safe and satisfactory by intraoperative fluoroscopy. For Group B, the time was recorded from the completion of bony structure exposure, ready to start placing the first pedicle screw, until the last pedicle screw was placed and the screw was confirmed to be safe and satisfactory by intraoperative fluoroscopy. According to the Neo grading standard (11): Grade 0 screws are all located inside the pedicle, without penetrating the cortex of the pedicle; Grade 1, the pedicle screw has penetrated the cortex, but <2 mm; Grade 2, the pedicle screw has penetrated the cortex >2 mm, but <4 mm; Grade 3, the pedicle screw has penetrated the cortex >4 mm. The calculation method for the placement satisfaction rate is: (Grade 0 + Grade 1) screw count/total screw count × 100%. The penetration rate calculation method is: (Grade 1 + Grade 2 + Grade 3) screw count/total screw count × 100%. Imaging observation indicators: Before surgery, 1 week after surgery, and at the last follow-up, the C2/3 angle, C2/3 displacement distance, and cervical lordosis angle were measured. Fracture healing and C2/3 fusion: The fracture and fusion conditions at various time points were observed. (1) Fracture healing criteria: The fracture line is completely closed with callus and bone trabeculae crossing the fracture line on cervical spine lateral x-ray films or cervical spine CT. (2) C2/3 facet joint fusion or intervertebral body fusion is defined as C2/3 fusion, and C2/3 fusion that occurs without bone grafting is called spontaneous fusion (12, 13). The criteria for facet joint fusion are: the disappearance of the facet joint space with continuous bone formation; the criteria for intervertebral body fusion (any of the following three conditions): (1) continuous bone bridge formation at the anterior edge of the vertebra; (2) continuous bone bridge formation at the posterior edge of the vertebra; (3) continuous bone bridge formation at both the anterior and posterior edges of the vertebra.

### 2.6 Statistical analysis

Data analysis was performed using SPSS 22.0 statistical software. Quantitative data were tested for normality using the Shapiro–Wilk test; if the data were normally distributed, they were expressed as the mean ± standard deviation. Inter-group comparisons were made using independent samples t-tests, while intra-group comparisons at different time points were made using paired t-tests. Comparisons between groups over time were conducted using repeated measures analysis of variance; if the sphericity test was not satisfied, the Greenhouse-Geisser method was used for correction. Multiple comparisons within the same group at different time points were performed using the Bonferroni method, and comparisons between different groups at the same time point were made using multivariate analysis of variance. For categorical data, inter-group comparisons were made using Fisher's exact probability method. Ordinal data inter-group comparisons were made using the non-parametric Wilcoxon rank-sum test. The significance level was set at a two-tailed *α* = 0.05.

## 3 Results

**Figure 1:**
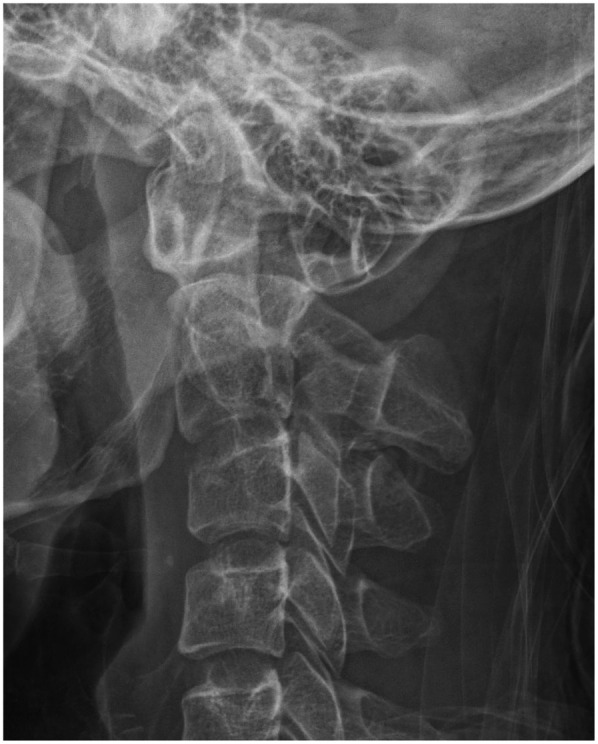
Preoperative lateral cervical radiographs. Preoperative cervical x-ray showed C2 Hangman fracture.

**Figure 2:**
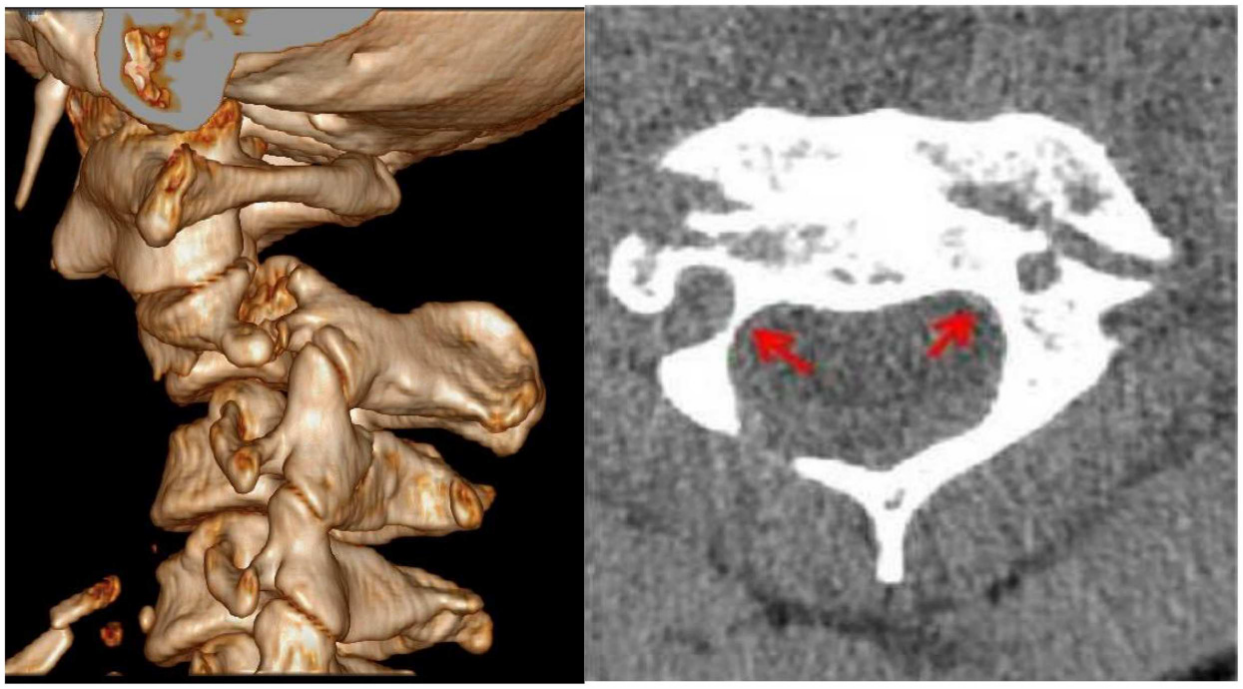
Preoperative cervical spine CT three-dimensional reconstruction and plain scan. The three-dimensional reconstruction showed Hangman fracture, and the axial view showed the high-riding vertebral artery, as a height of the isthmus internal marked by the left red arrow.

**Figure 3:**
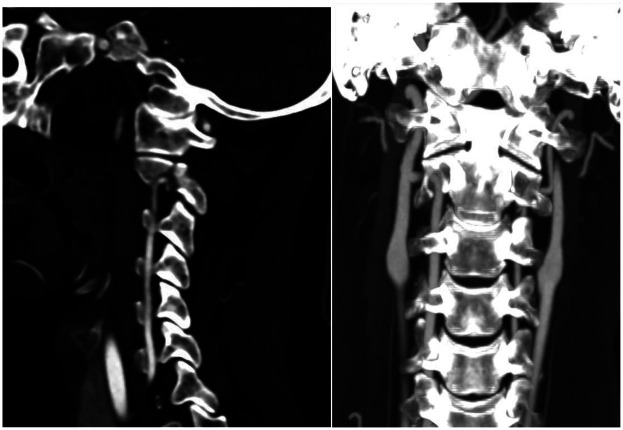
Preoperative cervical angiography CT sagittal and coronal reconstruction. Vertebral arteriography showed that the distance from the top of vertebral artery foramen to the upper articular surface was less than 2 mm, and the left side was the dominant artery.

**Figure 4:**
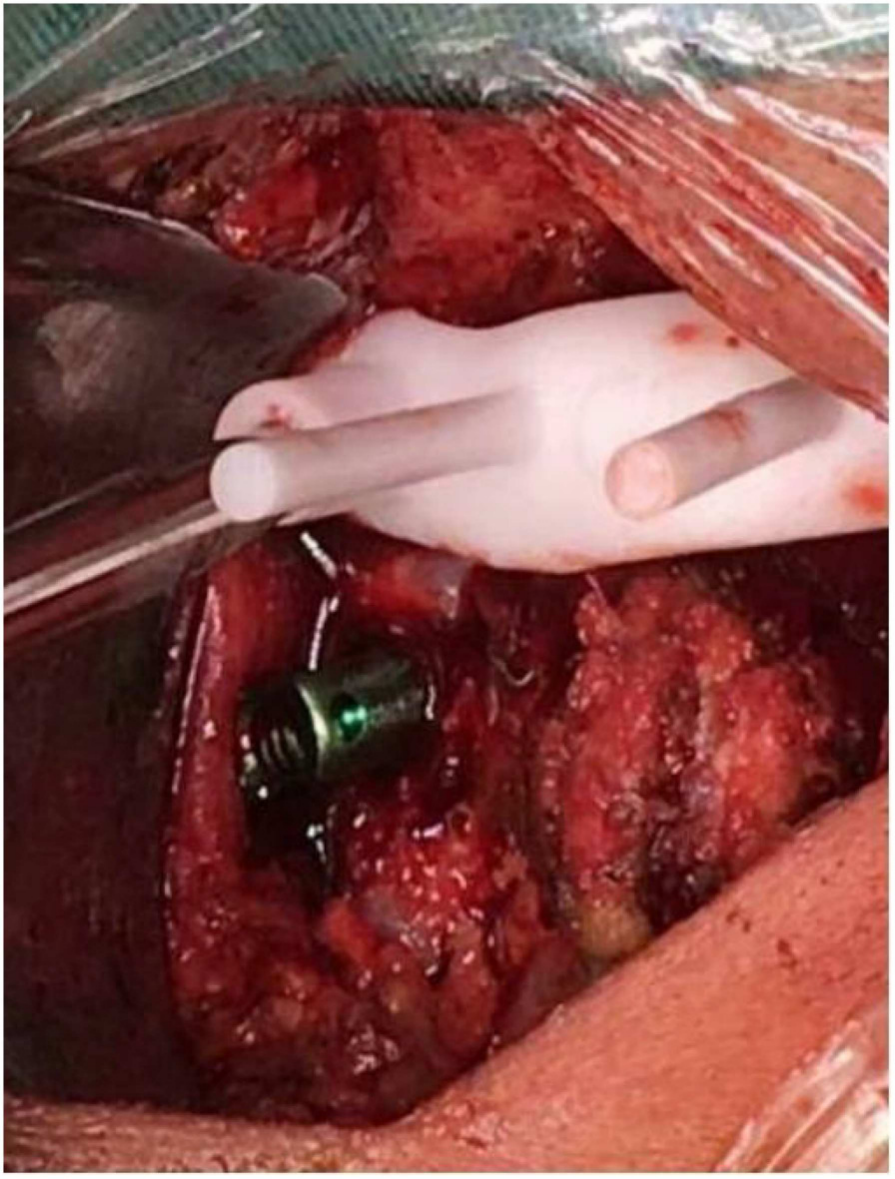
Intraoperative 3D-printed patient-specific templates guided pedicle screw placement. Intraoperative use of 3D printed patient specific templates for C1–2 pedicle screw placement.

**Figure 5:**
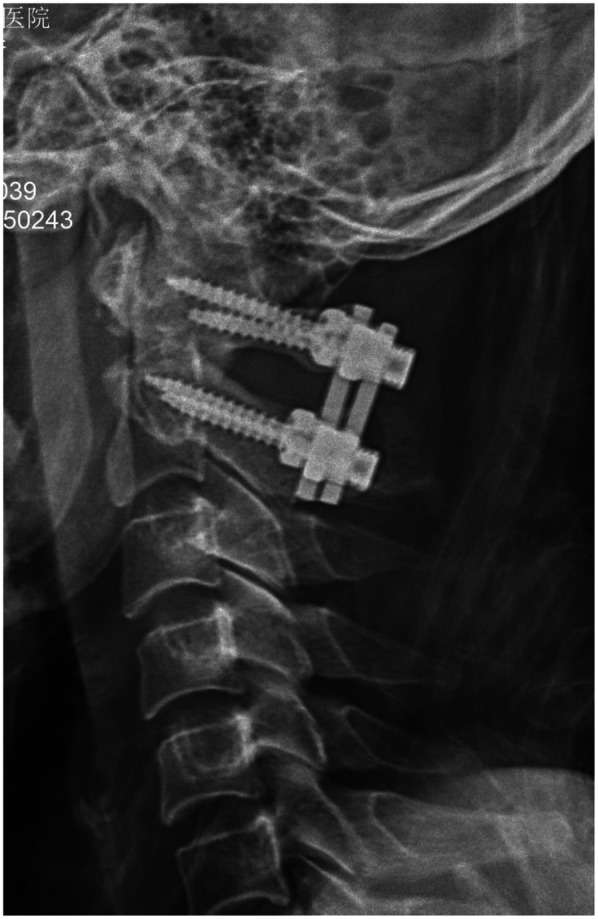
Lateral x-ray after pedicle screw fixation surgery. Postoperative x-ray showed fracture reduction.

All 33 patients successfully completed the surgery without vertebral artery, spinal cord, or major nerve injury during the operation. Typical cases are shown in Figures 1–5.

Patients were followed up for 12–48 months, with an average of (21.32 ± 9.54) months. The operation time and screws placement time in Group A were both greater than those in Group B(*P* < 0.05). The differences were 28.664 and 14.168, with 95% confidence intervals (12.107, 47.222) and (6.199, 19.137) respectively (*P* < 0.05). The intraoperative blood loss in Group A was greater than that in Group B(*P* < 0.05), with a difference of 47.483 and a 95% confidence interval of (18.761, 66.203) (*P* > 0.05). A total of 66 pedicle screws were placed in Group A, and 78 pedicle screws were placed in Group B. The screws placement satisfaction rate and cortical penetration rate in Group A were 98.48% and 6.06%, respectively, and in Group B were 98.72% and 6.41%, respectively (*P* > 0.05). The difference in screws placement satisfaction rate between Groups A and B was 0.246, with a 95% confidence interval of (0.114, 0.542) (*P* > 0.05). The difference in cortical penetration rate was 0.365, with a 95% confidence interval of (0.174, 1.023) (*P* > 0.05).There was no statistically significant difference in the proportion of pedicle screws and lamina screws between the two groups(*P* > 0.05). Both groups of patients had pedicle screws inserted for Type III fractures, with 8 screws placed in each group. The accuracy rate in Group A was 87.5%, and the penetration rate was 37.5%, while in Group B the accuracy rate was 100%, and the penetration rate was 12.5% (*P* < 0.05) (Table 3). The VAS scores and NDI scores of both groups at 1 week after surgery, 3 months after surgery, and at the final follow-up were significantly improved compared to before surgery, and the VAS and NDI scores at the final follow-up and 3 months after surgery were further improved compared to 1 week after surgery (*P* < 0.05); there were no significant differences in VAS scores and NDI between the two groups at each follow-up time point (*P* > 0.05). At the final follow-up, all patients had good fracture healing without internal fixation loosening or fracture. The C2/3 angle, displacement, and cervical lordosis angle of both groups at 1 week after surgery and at the final follow-up were significantly improved compared to before surgery (*P* < 0.05), and there were no significant differences between the two groups at the same time points (*P* > 0.05) (Table 2). The fusion and fracture healing conditions of C2/3 facet joints and intervertebral bodies at each follow-up time point are shown in Table 3. There were no significant differences in the fusion rate and fracture healing rate of C2/3 facet joints and intervertebral bodies between the two groups at each follow-up time point (*P* > 0.05). Templates for five randomly selected patients were re-printed one month later; superimposition revealed <0.2 mm translational and <1° angular difference vs. the first print [intra-class correlation coefficient (ICC) = 0.98].

**Table 2:** Comparison of operative time, blood loss, efficacy evaluation indexes and imaging measurement between the two groups.

Indicator	Group A (*n* = 15)	Group B (*n* = 18)	Statistical Value	*P*
Operative time (min)	123.15 ± 25.13	95.35 ± 16.23	*t* = 3.368	0.001
			MD = 28.664	0.000
			95% CI(12.107, 47.222)	
Screw insertion time (min)	46.34 ± 6.32	32.57 ± 5.34	*t* = 2.325	0.000
			MD = 14.168	0.000
			95% CI(6.199, 19.137)	
Intraoperation blood loss (ml)	152.15 ± 28.78	105.24 ± 18.54	*t* = 3.534	0.000
			*MD* = 47.483	0.000
			95% CI(18.761, 66.203)	
VAS score of neck pain				
Pre-operation	6.58 ± 1.83	6.63 ± 1.87	*t* = −0.262	0.841
1 week after surgery	3.41 ± 0.88*	3.44 ± 0.83*	*t* = −0.167	0.667
3 months after surgery	1.31 ± 0.36*^,^**	1.36 ± 0.31*^,^**	*t* = −0.285	0.831
Final follow-up	1.02 ± 0.25*^,^**	1.01 ± 0.23*^,^**	*t* = −0.198	0.722
NDI (%)				
Pre-operation	28.22 ± 3.12	28.54 ± 3.25	*t* = −0.312	0.876
1 week after surgery	21.46 ± 2.62*	21.38 ± 2.57*	*t* = −0.262	0.664
3 months after surgery	13.52 ± 2.13*^,^**	14.02 ± 2.31*^,^**	*t* = −0.124	0.433
Final follow-up	2.12 ± 1.02*^,^**	2.15 ± 1.01*^,^**	*t* = −0.112	0.312
Angulation of C2/3 (°)				
Pre-operation	12.21 ± 2.27	12.17 ± 2.36	*t* = −0.312	0.862
1 week after surgery	6.21 ± 1.02*	6.25 ± 1.04*	*t* = −0.161	0.537
Final follow-up	6.27 ± 1.08*^,^**	6.26 ± 1.08*^,^**	*t* = −0.135	0.331
Displacement of C2/3 (mm)				
Pre-operation	4.61 ± 1.25	4.53 ± 1.16	*t* = −0.434	0.661
1 week after surgery	1.23 ± 0.43*	1.22 ± 0.41*	*t* = −0.125	0.342
Final follow-up	1.26 ± 0.47*^,^***	1.23 ± 0.41*^,^***	*t* = −0.135	0.322
C2–C7 Cobb angle (°)				
Pre-operation	10.33 ± 2.27	10.62 ± 2.96	*t* = −0.731	0.732
1 week after surgery	12.86 ± 2.11*	13.17 ± 2.13*	*t* = −0.225	0.451
Final follow-up	13.12 ± 2.12*^,^***	13.22 ± 2.11*^,^***	*t* = −0.167	0.263

* Compared with preoperation, *P* < 0.05.

** Compared with postoperative 1 week, *P* < 0.05.

*** Compared with postoperative 1 week, *P* > 0.05.

**Table 3:** Comparison of screw accuracy and fusion between the two groups.

Indicator	Group A(*n* = 15)	Group B(*n* = 18)	Statistical Value	*P*
Total Number of Screws	66	78		
Pedicle screws	47 (71.21%)	58 (74.36%)	*x^2^* *=* 0.179	0.672
Lamina screws	19 (28.79%)	20 (25.64%)		
Grade 0	62	73	z = 0.216	0.642
Grade I	3	4		
Grade II	1	1		
Grade III	0	0		
Accuracy Rate	98.48%	98.72%	*OR* = 0.246	0.214
			95% CI (0.114, 0.542)	
Penetration Rate	6.06%	6.41%	*OR* = 0.365	0.187
			95% CI (0.174, 1.023)	
Type III fractures	3	3		
Pedicle screws	8	8		
Grade 0	5	7	z = 0.766	0.032
Grade I	2	1		
Grade II	1	0		
Grade III	0	0		
Accuracy Rate	87.50%	100.00%	*OR* = 12.500	0.000
Penetration Rate	37.50%	12.50%	*OR* = 37.500	0.000
Facet joints fusion			z = 0.443	0.865
Six months after surgery	9	10		
One year after surgery	14	16		
Final follow-up	15	18		
Interbody fusion			z = 0.672	0.872
Six months after surgery	2	2		
One year after surgery	5	6		
Final follow-up	7	7		
Fracture line healing			z = 0.376	0.631
Six months after surgery	11	14		
One year after surgery	13	17		
Final follow-up	15	18		

Two independent spinal surgeons (blinded) performed virtual screw placement on the same ten CT datasets; ICC for entry point and trajectory angle was 0.96 (intra-observer) and 0.94 (inter-observer).

## 4 Discussion

Hangman's fractures are classified into three types based on the mechanism of injury, fracture morphology, and degree of instability: Type I fractures refer to stable fractures with displacement <2 mm, which do not worsen in flexion and can be reduced in extension. Type II fractures refer to unstable fractures with C2/C3 displacement >2 mm, which may be associated with angulation. Type IIa fractures refer to unstable fractures with minimal or no C2/C3 displacement but significant angulation. Type III fractures refer to Hangman's fractures with C2/C3 facet joint dislocation and locking, which are severely unstable (17, 3). Simple C2 pedicle screw fixation can effectively treat unstable Hangman's fractures, with the advantage of physiological fixation, but the presence of vertebral artery anomalies increases the risk of spinal cord, nerve root, and vertebral artery injury during manual operation. High-riding vertebral artery anomalies lead to a reduction in the height and (or) width of the axis isthmus, meaning that the axis isthmus is partially occupied by the transverse foramen. When patients have a high-riding vertebral artery, it increases the difficulty of placing an axis pedicle screw. Research by Chun-Pi et al. (18) shows that up to 14.5% of patients have concurrent high-riding vertebral artery, and for patients with HRVA, imaging measurements show that the risk of vertebral artery injury during TA technique (Magerl screw) and Harms technique can be as high as 63% and 49%, respectively. Another study shows that 20% to 27% of patients with upper cervical spine diseases have high-riding vertebral artery, and if C2 pedicle screw fixation is performed at this time, the risk of vertebral artery injury due to pedicle screw penetration through the transverse foramen can be as high as 49% (19). If the vertebral artery is injured during surgery, it can lead to increased bleeding that is difficult to control and may cause irreversible damage to the spinal cord and nerves (20). To reduce the risk of vertebral artery injury during surgery, most scholars believe that for patients with high-riding vertebral artery, personalized treatment plans can be formulated before surgery, and appropriate internal fixation methods can be selected to improve the accuracy of screw placement and the cure rate of patients (21). Navigation technology-assisted pedicle screw placement, with multi-angle tomographic imaging reference, tries to select the screws channel that passes vertically through the fracture end at all angles, which has the advantages of accuracy, safety, and stability (22). However, this method necessitates intraoperative registration, which makes the procedure more cumbersome and thus increases both the surgical and anesthesia times. Moreover, due to the flexibility of the cervical spine, the spatial orientation captured by CT scanning during surgery often deviates from the actual spatial orientation. This deviation can lead to navigation defocus, failure in screws placement, and even severe nerve and vascular injuries. Additionally, obstruction by soft tissues can hinder the flexible and free use of surgical instruments, resulting in deviations during screw placement. Especially in patients with Type III fractures, the accuracy rate of navigation-assisted screw placement is significantly lower than that of the 3D printed navigation template group, while the penetration rate is the opposite, indicating that the use of navigation technology should be approached with caution for particularly unstable fractures. This is because cervical unstable fractures exhibit significant spatial position changes under the influence of external forces, leading to navigation drift. With the combination of digital medicine and orthopedics, 3D printing technology has gradually been applied to spinal orthopedics. The application of 3D printed navigation templates in pedicle screw fixation has the characteristics of optimizing preoperative planning, providing personalized and precise treatment, and can effectively avoid the obstruction of high-riding vertebral artery on screws placement, reducing the difficulty of screws placement and improving the accuracy of screws placement, and avoiding vertebral artery injury. For instance, preoperatively, with the aid of computer design, the screws channel can be accurately designed to pass through the fracture line of Hangman's fracture, calculate the specifications of the proposed screw, design the screws angle and depth, and observe the three-dimensional course of the vertebral artery, designing a screws channel to avoid the vertebral artery; simulation of screws placement can increase the proficiency of the operator, and at the same time, it can preview the key areas and difficulties of screws placement, playing a role in reducing the risk of spinal cord and vertebral artery injury during screws placement; during screws placement, it can be compared and observed in real-time with the 3D model, clarifying the anatomical structure and adjacent relationships, reducing judgment errors and screws-related complications caused by vertebral artery variation; the navigation template has good matching with the screws segment, and screws placement can be guided by pasting on the bony structure of the vertebral plate surface, reducing the number of secondary screws placements, reducing the difficulty and risk of screws placement; after screws placement, only one verification fluoroscopy is needed, greatly shortening the screws placement time, reducing the number of x-ray fluoroscopies and screws placement bleeding. This study's results show that patients in the 3D printed navigation template group had significantly shorter surgical times, screws placement times, and intraoperative blood loss compared to the intraoperative navigation group, thus it can be considered that the 3D printed navigation template has excellent safety. There was no significant difference in screws placement satisfaction rate and screws penetration rate between the intraoperative navigation group and the 3D printed navigation template group, directly proving the accuracy of the 3D printed navigation template. Both groups of patients underwent placement of laminar screws, and the usage ratio was not different, proving that the efficiency of 3D printed navigation template-guided screw placement is not inferior to navigation technology. 3D printed navigation templates can expand the scope of use in cases with high-riding vertebral arteries. The volume and surface area projection of the 3D printed navigation template are not larger than the laminar area, hence there is no need for excessive exposure of the outer side of the lamina or the C2 spinous process, which will not lead to excessive transection of muscle attachment points. In our experience, this does not result in axial symptoms or cervical kyphosis. On the contrary, navigation technology requires the placement of reference frames and excessive stripping of the muscle attachment points on the spinous process. Moreover, to avoid muscle obstruction of the navigation handle, excessive lateral exposure is needed. In this study, all 33 patients successfully completed the surgery, with no vertebral artery, spinal cord, or major nerve injury during the operation. The screws placement satisfaction rate was higher than 98%. VAS scores and NDI scores were significantly increased compared to before surgery. The C2/3 angle, displacement, and cervical lordosis angle were significantly improved compared to before surgery. Fracture healing was satisfactory. Patients who underwent navigation-guided screws placement during surgery had higher intraoperative blood loss and operation time than those who underwent 3D printed navigation template-guided screws placement.

The limitations of this study include a small sample size; with an increased sample size, more statistically significant conclusions may be drawn. Multicenter trials need to be added to verify the results and explore the long-term fusion rate. Further analysis and research are needed after collecting and analyzing more cases. The application of 3D printing technology still has some drawbacks, such as the high cost of 3D printing equipment, limited choices of printing materials, and the time-consuming process from data acquisition, modeling, design, printing, to sterilization, as well as high economic costs. Further research is needed to improve the design of the guide template and optimize the manufacturing and usage processes to reduce costs and promote wider adoption. All procedures were performed by experienced surgeons, limiting generalizability to less specialized centers.

## 5 Conclusion

In conclusion, our study highlights the efficacy of 3D printed navigation templates in enhancing the precision and safety of pedicle screw placement in upper cervical spine surgeries, particularly in complex anatomical scenarios such as Hangman's fractures with HRVA. The technique not only matches the accuracy of traditional navigation systems but also provides the advantage of reduced surgical duration and blood loss. Importantly, the use of 3D printed guides may mitigate the risk of vertebral artery injury, a critical concern in cervical spine surgeries. Compared to navigation technology, it can effectively improve the accuracy of screw placement in extremely unstable fractures. These findings underscore the potential of 3D printing technology as a valuable adjunct in preoperative planning and intraoperative guidance, contributing to improved patient outcomes in spinal surgery. Future studies with larger cohorts may further validate these preliminary yet promising results.

All data generated or analysed during this study are included in this published article and its Supplementary information files.

## Data Availability

The original contributions presented in the study are included in the article/Supplementary Material, further inquiries can be directed to the corresponding author.
